# A New Scale to Assess the Severity and Prognosis of Pulmonary Alveolar Proteinosis

**DOI:** 10.1155/2016/3412836

**Published:** 2016-08-18

**Authors:** JiuWu Bai, JinFu Xu, WenLan Yang, Beilan Gao, Weijun Cao, Shuo Liang, Huiping Li

**Affiliations:** ^1^Department of Respiratory Medicine, Shanghai Pulmonary Hospital, Tongji University School of Medicine, 507 Zheng Min Road, Shanghai 200433, China; ^2^Department of Pulmonary Function Test, Shanghai Pulmonary Hospital, Tongji University School of Medicine, 507 Zheng Min Road, Shanghai 200433, China

## Abstract

*Background*. Pulmonary Alveolar Proteinosis (PAP) is a syndrome characterized by pulmonary surfactant accumulation. Small proportion of PAP patients experienced spontaneous remission.* Objective*. The aim of this study was to assess the severity and prognosis of PAP using various indexes.* Methods*. Characteristics, PaO_2_, lung function parameters, and HRCT score of 101 patients with PAP were retrospectively analyzed. Many indexes were explored and integrated into a scale.* Results*. PaO_2_ was lower among smokers than among never-smokers. PaO_2_ differed between each pair of patient groups stratified according to HRCT score or DLCO, % predicted, which differed between any two groups stratified according to PaO_2_. The PAP patients who died presented with more symptoms, a higher HRCT score, and lower DLCO, % predicted, than survivors. Smoking status, symptoms, PaO_2_, HRCT score, and DLCO, % predicted, were integrated into a scale (severity and prognosis score of PAP (SPSP)). SPSP correlated positively with PaO_2_, FVC, % predicted, FEV_1_, % predicted, and DLCO, % predicted, and negatively with HRCT score. The patients who died displayed a higher SPSP than survivors.* Conclusion*. Smoking status, symptoms, PaO_2_, HRCT score, and DLCO, % predicted, were integrated into a scale (SPSP) that can be used to assess the severity and prognosis of PAP to some degree.

## 1. Introduction

Pulmonary Alveolar Proteinosis (PAP) is a rare lung syndrome characterized by the intra-alveolar accumulation of surfactant lipids and proteins, which impairs gas exchange and results in progressive respiratory insufficiency. PAP was first described in 1958 [1] and was divided into three subtypes by Carey and Trapnell: congenital PAP, secondary PAP, and autoimmune PAP [2]. Autoimmune PAP is a disorder of unknown etiology and accounts for approximately 90% of all PAP cases. Autoimmune PAP was first confirmed by Tanaka et al. to be an autoimmune disease using a neutralizing antibody of immunoglobulin G isotype against granulocyte/macrophage colony-stimulating factor (GM-CSF) [3]. Surfactant proteins are primarily cleared by alveolar type II epithelial cells and alveolar macrophages. GM-CSF can bind to receptors on the surface of alveolar macrophages, thus promoting the removal of surfactant proteins via PU.1 activity [4]. The levels of anti-GM-CSF antibodies are significantly increased in serum and bronchoalveolar lavage fluid (BALF) of patients with autoimmune PAP [5], and these antibodies display high affinity for GM-CSF and decrease GM-CSF activity [6]. One report indicated that injecting human anti-GM-CSF antibodies into nonhuman primates may induce the occurrence of autoimmune PAP [7].

The present therapeutic methods for PAP include whole lung lavage (WLL), subcutaneous or inhaled GM-CSF, rituximab, plasmapheresis, and lung transplantation [8]. One report indicated that a small proportion of PAP patients experienced spontaneous remission [9]. The disease severity score (DSS), which is based on the presence of symptoms and the degree of reduction in PaO_2_, was suggested as an index of the severity of PAP and was divided into 5 grades by Inoue et al. [10]. But the degree of shadowing in chest images did not correspond to the degree of symptoms in certain clinical cases. Whether DSS can predict the prognosis of PAP has not been reported. The aim of the current study was to explore various indexes associated with the severity and prognosis of PAP by analyzing epidemiologic, clinical, and laboratory features of PAP.

## 2. Methods

### 2.1. Study Population

**Figure 1:**
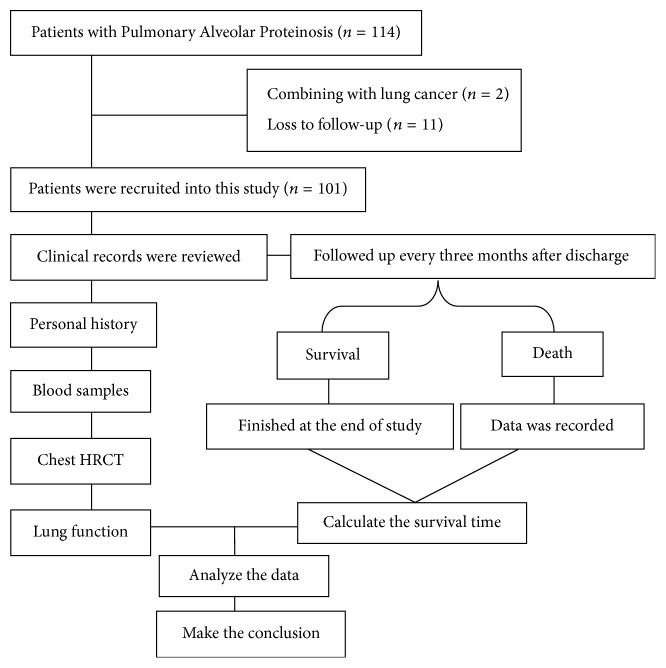
The detailed procedure of screening patients who were recruited to participate in this study.

This study was conducted in Shanghai Pulmonary Hospital affiliated to Tongji University in China and consisted of a retrospective cross-sectional analysis up to 2015. Between January 2004 and July 2015, 114 patients were diagnosed with PAP in our institution. Among these patients, 2 had comorbid lung cancer, and 11 were lost to follow-up. The remaining 101 PAP patients were enrolled in this study (Figure 1). All patients included in the retrospective aspect of this study received follow-up phone calls to ensure participation. Written informed consent was obtained from all of the patients. The Ethics Committee of Shanghai Pulmonary Hospital approved the study protocol (K15-185).

### 2.2. Diagnostic Criteria

Eligibility criteria, which were selected as described by Ben-Dov and Segel [11], included histopathologic findings of specimens obtained by open lung biopsy or transbronchial lung biopsy; a milk-like appearance with typical cytological findings and lamellar bodies of BALF on electron microscopy; ground glass opacity and/or a crazy paving pattern on high resolution computed tomography (HRCT); restrictive ventilation and diffusion dysfunction; hypoxemia; dyspnea and cough. A small proportion of patients were asymptomatic at diagnosis. In this study, a diagnosis of PAP was established by characteristic HRCT findings in the chest and in BALF (*n* = 43), transbronchial lung biopsy results (*n* = 20), or open lung biopsy results (*n* = 38).

### 2.3. Interview Questionnaire and Blood Samples

A standardized protocol was used to obtain informed consent from each subject during a medical visit. The interview questionnaire that was used included questions on the following topics: general and anthropometric information (i.e., age and sex); smoking history (e.g., smoker, ex-smoker, or never-smoker); history of occupational exposure (e.g., dust, fume, and grease); and clinical manifestation (e.g., the onset of symptoms and the course of disease).

### 2.4. Grading of Chest HRCT Scans

HRCT scans of the chest of 101 patients were analyzed and graded according to the visual scoring methods proposed by Lee et al. [12]. The chest HRCT was examined and interpreted independently by two chest physicians. The mean values obtained from the two readers were used for analysis. We selected the HCRT grades in four representative regions: the aortic arch, the tracheal carina, and the convergence of the left and right inferior lung veins and above the diaphragm. “Ground glass opacity” refers to the presence of increased lung opacity associated with partial obscuring of normal vascular structures. The extent of lung opacity was estimated using a five-point scale: no opacity, 0; opacity involving <25% of a region of hemithorax, 1; 25–50%, 2; 50–75%, 3; and ≥75%, 4. The chest HRCT score was calculated by summing the lung opacity scores of the four representative regions of each hemithorax.

### 2.5. Pulmonary Function Assessment

The data collected included FVC, FEV_1_, FEV_1_/FVC, diffusing capacity of the lung for carbon monoxide (DLCO), and arterial blood gases. The FVC, FEV_1_, and DLCO data were presented as the percentages of predicted values (% predicted). Arterial blood measurements were performed on samples obtained while the patient was breathing room air at rest in the supine position. PaO_2_ was the main parameter analyzed.

### 2.6. Survival Analysis

In our department, all of the patients were routinely asked to sign a consent form when they were admitted to the hospital. Patients signed the consent form to authorize follow-up every 3 months through telephone or face-to-face interviews. The follow-up was completed on October 31, 2015. A patient was considered lost to follow-up if we were unable to contact him/her at each follow-up session during the study period. The endpoint of this study was all-cause mortality. Information regarding the cause and date of death was obtained from hospital medical records if the patient died in the hospital or from official death certificates in other circumstances.

### 2.7. Statistics

SPSS version 19.0 (SPSS, Chicago, Illinois) was used for statistical analysis. The data were tabulated as the means and standard deviations for quantitative variables or as absolute numbers and percentages for qualitative variables. The Kolmogorov-Smirnov test was used to analyze the data distribution for each variable. PaO_2_ of patients with PAP was comparatively analyzed between groups stratified according to age, sex, symptoms, smoking status, occupational exposure, HRCT score, and lung function. The correlations of selected indexes (i.e., HRCT score, FVC, % predicted, FEV_1_, % predicted, and DLCO, % predicted) with PaO_2_ were also analyzed. Those indexes which were associated with PAP severity and prognosis were integrated into a scale. In the bivariate analysis, Student's *t* -test for independent variables was used to analyze variables that were normally distributed, and the Mann-Whitney *U*  test was used to analyze variables that were nonnormally distributed. Qualitative variables were compared using the chi-square test. The variables that presented statistically significant differences (*P* < 0.05) based on the bivariate analysis and that were of clinical interest were included as independent variables in the initial model. Then, a forward stepwise technique (i.e., the Wald test) was used to remove the variables that displayed a *P* > 0.1 from the final model. *P* ≤ 0.05 was considered indicate a significant difference.

## 3. Results

### 3.1. Demographics

Men accounted for more than two-thirds of the patients with PAP (Table 1). There was no apparent difference in PaO_2_ between men and women (Table 1). The median age at diagnosis was 49 years. There was no apparent difference in PaO_2_ between age groups (≤50 years versus >50 years) (Table 1).

A history of smoking was reported in 42 (41.6%) patients, all of whom were men. PaO_2_ of smokers (including ex-smokers) was lower than that of never-smokers (*P* = 0.035) (Table 1). Approximately half of all patients had a history of occupational exposure. Four-fifths of the patients were symptomatic at diagnosis. There was no apparent difference in PaO_2_ between those with and without a history of occupational exposure and between those presenting with and without symptoms (Table 1).

**Figure 2:**
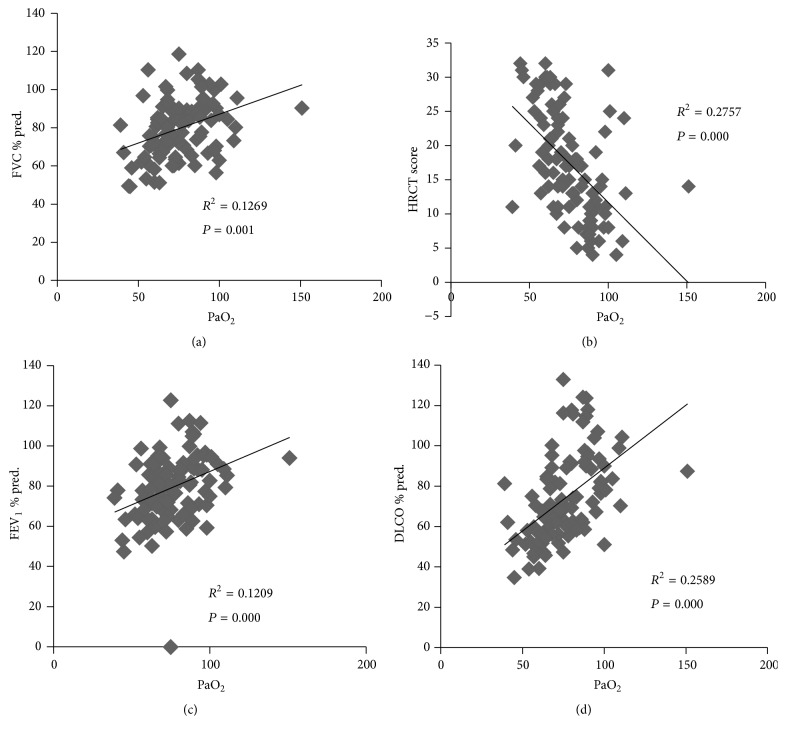
(a) Scatter diagram of PaO_2_ with HRCT score. (b) Scatter diagram of PaO_2_ with FVC, % predicted. (c) Scatter diagram of PaO_2_ with FEV_1_, % predicted. (d) Scatter diagram of PaO_2_ with DLCO, % predicted.

PaO_2_ positively correlated with FVC, % predicted, FEV_1_, % predicted, and DLCO, % predicted (*r* = 0.330, 0.361, and 0.509, all *P* < 0.01), and negatively correlated with HRCT score (*r* = −0.525, *P* < 0.01) (Figure 2). The correlation of DLCO, % predicted, with PaO_2_ was the strongest among the three lung function indexes and was regarded as the main indicator of lung function. Next, the patients were divided into three groups based on DLCO, % predicted (≥80, 60–80, and <60). Differences in PaO_2_ were detected between each pair of groups stratified according to DLCO, % predicted, groups (all *P* < 0.05) (Table 2).

Then, the patients were divided into four groups according to HRCT score (≤8, 8–16, 16–24, and 24–32) (Table 3). Differences in PaO_2_ were observed between each pair of groups stratified according to HRCT score (all *P* < 0.05) (Table 3).

Alternatively, the patients were divided into three groups according to PaO_2_ (≥80 mmHg, 60–80 mmHg, and <60 mmHg). Differences in HRCT score and DLCO, % predicted, were detected between each pair of groups stratified according to PaO_2_ (all *P* < 0.05) (Table 4).

The differential characteristics of the group of survivors throughout the follow-up period (*n* = 94) and the group of nonsurvivors (*n* = 7) are shown in Table 5. The patients who ultimately died presented with more symptoms, a higher HRCT score, and lower FVC, % predicted, FEV_1_, % predicted, and DLCO, % predicted, than the patients who survived.

Smoking status, PaO_2_, HRCT score, and DLCO, % predicted, were at least partially associated with the severity of PAP. Symptoms, HRCT score, and DLCO, % predicted, were associated with PAP patient prognosis to some degree. Smoking status, symptoms, PaO_2_, HRCT score, and DLCO, % predicted, were integrated into a scale (severity and prognosis score of PAP (SPSP)) of Arabic numerals as a measure of the severity and prognosis of PAP (Table 6). Similar to DSS, SPSP positively correlated with HRCT score and negatively correlated with PaO_2_, FVC, % predicted, FEV_1_, % predicted, and DLCO, % predicted (all *P* < 0.05). The absolute “*r*” values for the correlations of SPSP with HRCT score, FVC, % predicted, FEV_1_, % predicted, and DLCO, % predicted, were higher than those for the correlations of DSS with these indexes, except that the absolute “*r*” value for the correlation of SPSP with PaO_2_ was similar to that for the correlation of DSS with PaO_2_ (Table 7). The patients who ultimately died displayed a higher SPSP than the patients who survived (*P* < 0.05) (Table 8). Therefore, PAP patient mortality increased as SPSP increased (Table 9).

## 4. Discussion

DSS, consisting of the combination of symptoms and PaO_2_, was developed by Inoue et al. [10]. The basis of this study was that PaO_2_ can reflect the severity of this disease. In this study, the median age at diagnosis was 49 years, similar to that of the study in Japan by Inoue et al. (51 years) [10] and far greater than that of the study by Seymour and Presneill (39 years) [13]. Whether race is associated with PAP requires further investigation. The similarity of PaO_2_ between age groups implied that age is not associated with the severity of PAP. The ratio of males to females in this study was 2.48 : 1; similar to the values reported previously [9, 10, 13]. The apparent lack of a difference in PaO_2_ between males and females indicated that sex is not associated with the severity of PAP. Age and sex were not associated with PAP patient prognosis based on the finding that survivors and nonsurvivors displayed similar results for these two characteristics.

Analysis of smoking status suggested that 58.3% of the male patients had a history of smoking but that none of the female patients had a history of smoking; these rates were lower than those in a previous report (74% male and 8.5% female) [10]. In this study, PaO_2_ of smokers (including ex-smokers) was lower than that of never-smokers. One previous report suggested that the number of WLLs necessary to reach remission was higher for smokers than for never-smokers [14]. Those results indicated that smoking is an important factor associated with the severity and prognosis of PAP. The percentages of PAP patients who experienced occupational exposure were 55.6% of males and 34.5% of females, and these values were higher than those previously reported (32% of males and 13% of females) [10]. Cummings et al. reported 2 patients with PAP who had contacted indium tin oxide, and a high serum anti-GM-CSF antibody level was found in 1 of these patients [15]. However, there was no apparent difference in PaO_2_ between those with and without occupational exposure. The total percentages of patients who had a history of smoking and/or occupational exposure were 79.2% of males and 34.5% of females. This result indicated that smoking and occupational exposure may be very important influencing factors for PAP, and the difference in this percentage between males and females is likely related to variations in professions and habits between sexes.

A total of 86.1% of PAP patients were symptomatic; this value was higher than that previously reported by Inoue et al. (68.4%) [10]. However, no significant difference in PaO_2_ was detected between symptomatic and asymptomatic PAP patients. This result indicated that the presence of symptoms was insufficient as an index of PAP severity. Despite this finding, the patients who ultimately died presented with more symptoms than the patients who survived; therefore, the presence of symptoms was associated with PAP patient prognosis.

PaO_2_ correlated with HRCT score, FVC, % predicted, FEV_1_, % predicted, and DLCO, % predicted. PaO_2_ was adopted as the main factor in DSS by Inoue et al. [10]. The HRCT score reflects the degree of shadowing. In this study, the patients were separated into four groups according to HRCT score. PaO_2_ differed between each pair of groups stratified according to HRCT score. The HRCT score partially reflects the severity of PAP. Restrictive ventilatory dysfunction is always observed in PAP. The “*r*” value for the correlation of PaO_2_ with DLCO, % predicted, was the greatest among the three lung function parameters. In this study, an apparent difference in PaO_2_ was observed between each pair of groups stratified according to DLCO, % predicted (≥80, 60–80, and <60). Furthermore, HRCT score and DLCO, % predicted, differed between each pair of groups stratified according to PaO_2_ (≥80 mmHg, 60–80 mmHg, and <60 mmHg). PaO_2_, HRCT score, and DLCO, % predicted, were associated with the severity of PAP. The patients who ultimately died displayed a higher HRCT score and a lower DLCO, % predicted, than the patients who survived. In contrast, PaO_2_ was not different between the patients who died and the patients who survived.

Smoking status, symptoms, PaO_2_, HRCT score, and DLCO, % predicted, were integrated into a scale (SPSP) that was used as a measure of PAP severity and patient prognosis. SPSP, which ranged from 1 to 10, displayed stronger correlations with FVC, % predicted, FEV_1_, % predicted, DLCO, % predicted, and HRCT score than DSS. The patients who died displayed a higher SPSP than the patients who survived. PAP patient mortality increased as SPSP increased. Therefore, SPSP reflects PAP severity and predicts PAP patient prognosis to some degree.

Considering the differential prognosis of PAP patients based on SPSP, we propose the following advice. If SPSP ≤ 2, the patient with autoimmune PAP should quit smoking, refrain from occupational exposure, and undergo follow-up assessments consisting of arterial blood gas analyses, lung function tests, and chest HRCT every 3–6 months; the cause of secondary PAP is explored and removed. If SPSP > 2 to ≤4, the time interval of follow-up visit can be shortened according to the disease evolution. If SPSP > 4, the patient with autoimmune PAP should begin treatment with WLL or inhaled GM-CSF, while the patient with secondary PAP is only treated through WLL on the basis of treating primary diseases. WLL remains the first-line standard for the treatment of PAP [16]. If WLL treatment fails, inhaled GM-CSF is the next treatment to be attempted. The results of a meta-analysis of the therapeutic efficacy of GM-CSF for PAP showed that 76.5% and 43% of PAP patients using inhaled and subcutaneous GM-CSF, respectively, responded to treatment [17]. In addition, the anti-CD-20 antibody rituximab is another promising therapy for PAP [18].

The main limitation of our study was that the number of patients who died was limited. Furthermore, the disease type was not further distinguished between autoimmune PAP and secondary PAP. However, because it was reported that autoimmune PAP accounts for approximately 90% of all PAP cases, the results of this study still have considerable clinical value. The SPSP is a new scale that must be verified by additional clinical data and be further optimized.

## 5. Conclusions

In this study, analyses of demographic characteristics, PaO_2_, HRCT score, and lung function parameters of 101 patients revealed that smoking status, PaO_2_, HRCT score, and DLCO, % predicted, were associated with PAP severity to some extent; additionally, symptoms, HRCT score, and DLCO, % predicted, were associated with PAP patient prognosis to some degree. Smoking status, symptoms, PaO_2_, HRCT score, and DLCO, % predicted, were integrated into a scale (SPSP) reflecting PAP severity and patient prognosis. Thus, SPSP can be used to assess PAP severity and predict PAP patient prognosis to some degree.

## Figures and Tables

**Table 1:** Demographic and clinical characteristics of the patients with PAP.

	*n*	%	Mean ± SD	PaO_2_, mmHg
Age, y	101		48.9 ± 11.2	
Age at diagnosis, y	101		47.4 ± 11.0	
≤50	62	61.4	38.5 ± 7.6	76.8 ± 17.6
>50	39	38.6	57.7 ± 5.4	74.8 ± 18.3
*P* value (≤50 versus >50)				0.581
Sex				
Male	72	71.3		75.9 ± 18.0
History of smoking/occupational exposure	42/40	58.3/55.6		
Neither history of smoking nor occupational exposure	15	20.8		
Female	29	28.7		76.2 ± 17.7
History of smoking/occupational exposure	0/10	0/34.5		
Neither history of smoking nor occupational exposure	19	65.5		
*P* value (male versus female)				0.954
Symptoms				
Yes	87	86.1		74.8 ± 16.2
No	14	13.9		79.4 ± 15.5
*P* value				0.317
Smoking status				
Smoker (including ex-smoker)	42 (8)	41.6 (7.9)		71.6 ± 13.9
Never-smoker	59	58.4		79.2 ± 19.6
*P* value				**0.035**
Occupational exposure				
Yes	50	49.5		77.1 ± 18.7
No	51	50.5		74.9 ± 16.9
*P* value				0.537

Data are presented as *n* (%) or mean ± SD unless otherwise stated. PaO_2_: arterial partial pressure of oxygen; PAP: Pulmonary Alveolar Proteinosis.

**Table 2:** Comparison of PaO_2_ values between different groups according to DLCO, % predicted.

DLCO, % predicted	*n*	%	PaO_2_, mmHg
≥80	37	36.6	86.3 ± 18.0
60–80	32	31.7	76.2 ± 14.5
<60	32	31.7	64.0 ± 12.6
*P* value			
≥80 versus 60–80			**0.013**
60–80 versus <60			**0.001**
≥80 versus <60			**<0.001**

Data are presented as *n* (%) or mean ± SD unless otherwise stated. DLCO: single-breath diffusing capacity of carbon monoxide; PaO_2_: arterial partial pressure of oxygen.

**Table 3:** Comparison of PaO_2_ values between different groups according to HRCT score.

HRCT score	*n*	%	PaO_2_, mmHg
Total	101		76.0 ± 17.8
≤8	17	16.8	90.5 ± 9.2
8–16	32	31.7	81.2 ± 19.6
16–24	30	29.7	71.3 ± 13.1
24–32	22	21.8	63.0 ± 14.5
*P* value			
≤8 versus 8–16			**0.029**
≤8 versus 16–24			**<0.001**
≤8 versus 24–32			**<0.001**
8–16 versus 16–24			**0.032**
8–16 versus 24–32			**0.001**
16–24 versus 24–32			**0.029**

Data are presented as *n* (%) or mean ± SD unless otherwise stated. HRCT: high resolution computed tomography; PaO_2_: arterial partial pressure of oxygen.

**Table 4:** Comparison of HRCT score and lung function values between different groups according to PaO_2_.

PaO_2_, mmHg	*n*	%	HRCT score	DLCO, % predicted
≥80	40	36.6	86.3 ± 18.0	11.4 ± 5.0
60–80	46	31.7	76.2 ± 14.5	17.3 ± 5.9
<60	15	31.7	64.0 ± 12.6	23.8 ± 6.9
*P* value				
≥80 versus 60–80			**<0.001**	**<0.001**
60–80 versus <60			**0.035**	**0.016**
≥80 versus <60			**<0.001**	**<0.001**

Data are presented as *n* (%) or mean ± SD unless otherwise stated. DLCO: single-breath diffusing capacity of carbon monoxide; HRCT: high resolution computed tomography; PaO_2_: arterial partial pressure of oxygen.

**Table 5:** The characteristics of the groups of survivors and nonsurvivors.

Parameter	All patients	Nonsurvivors	Survivors	*P* value
Subjects, number	101	7	94	
Sex, M/F, number	71/30	5/2	66/28	0.980
Age, y	48.7 ± 11.2	54.4 ± 9.9	48.3 ± 11.2	0.174
Smoking history, number (%)	42 (41.6%)	3 (42.9%)	39 (41.4%)	0.944
Occupational exposure, number (%)	50 (49.5%)	4 (57.1%)	46 (48.9%)	0.842
Onset of symptoms, y	47.3 ± 11.0	53.0 ± 9.3	46.8 ± 11.0	0.165
Course of disease, m	19.8 ± 33.3	19.7 ± 15.6	19.8 ± 34.4	0.997
Symptoms, number (%)	87 (86.1%)	7 (100%)	80 (85.1%)	**<0.001**
PaO_2_, mmHg	75.8 ± 17.7	66.4 ± 21.0	76.5 ± 17.4	0.127
HRCT score	17.3 ± 7.8	24.7 ± 7.3	16.7 ± 7.6	**0.008**
FVC, % predicted	80.0 ± 15.2	63.2 ± 13.8	81.3 ± 14.6	**0.002**
FEV_1_, % predicted	80.2 ± 15.1	63.3 ± 14.2	81.5 ± 14.5	**0.002**
FEV_1_/FVC	84.7 ± 6.4	89.1 ± 3.4	84.4 ± 6.5	0.058
DLCO, % predicted	73.9 ± 21.9	52.9 ± 10.9	75.5 ± 21.7	**0.001**

Data are presented as *n* (%) or mean ± SD unless otherwise stated. DLCO: single-breath diffusing capacity of carbon monoxide; FEV_1_: forced expiratory volume in 1 second; FVC: forced vital capacity; HRCT: high resolution computed tomography; PaO_2_: arterial partial pressure of oxygen.

**Table 6:** Severity and prognosis score of PAP (SPSP).

Severity criteria	Score
Smoking status	
Never-smoker	0
Smoker	1
Symptoms	
No	0
Yes	1
PaO_2_, mmHg	
≥80	0
60–80	1
<60	2
HRCT score	
≤8	1
8–16	2
16–24	3
24–32	4
DLCO, % predicted	
≥80	0
60–80	1
<60	2

Data are presented as *n* (%) or mean ± SD unless otherwise stated. DLCO: single-breath diffusing capacity of carbon monoxide; HRCT: high resolution computed tomography; PaO_2_: arterial partial pressure of oxygen; PAP: Pulmonary Alveolar Proteinosis; SPSP: severity and prognosis score of PAP.

**Table 7:** Relationships between SPSP or DSS and other indexes.

Characteristic	SPSP		DSS	
	*r*	*P* value	*r*	*P* value
PaO_2_, mmHg	0.778	**<0.001**	0.799	**<0.001**
HRCT score	0.846	**<0.001**	0.527	**<0.001**
FVC, % predicted	0.575	**<0.001**	0.332	**0.001**
FEV_1_, % predicted	0.557	**<0.001**	0.329	**0.001**
DLCO, % predicted	0.794	**<0.001**	0.513	**<0.001**

Data are presented as *n* (%) or mean ± SD unless otherwise stated. DLCO: single-breath diffusing capacity of carbon monoxide; DSS: disease severity score; FEV_1_: forced expiratory volume in 1 second; FVC: forced vital capacity; HRCT: high resolution computed tomography; PaO_2_: arterial partial pressure of oxygen; PAP: Pulmonary Alveolar Proteinosis; SPSP: severity and prognosis score of PAP.

**Table 8:** SPSP and DSS of patients according to outcome.

Parameter	All patients	Nonsurvivors	Survivors	*P* value
DSS	2.5 ± 1.0	3.3 ± 1.4	2.4 ± 0.9	0.157
SPSP	5.5 ± 2.3	7.9 ± 1.9	5.3 ± 2.3	**0.005**

Data are presented as *n* (%) or mean ± SD unless otherwise stated. DSS: disease severity score; SPSP: severity and prognosis score of PAP.

**Table 9:** Prognosis of patients according to SPSP.

	Severity and prognosis score of PAP (SPSP)				
	1 or 2	3 or 4	5 or 6	7 or 8	9 or 10
*n* (%)	13 (12.9%)	21 (20.8%)	32 (31.7%)	25 (24.8%)	10 (9.9%)
Prognosis					
Improvement, number (%)	10 (76.9%)	16 (76.2%)	25 (78.1)	20 (80%)	5 (50%)
Stable, number (%)	3 (23.1%)	4 (19.0%)	5 (15.6)	3 (12%)	1 (10%)
Exacerbation, number (%)	0 (0)	1 (4.8)	0 (0)	0 (0)	1 (10%)
Death, number (%)	0 (0)	0 (0)	2 (6.3)	2 (8%)	3 (30%)

Data are presented as *n* (%) or mean ± SD unless otherwise stated. PAP: Pulmonary Alveolar Proteinosis; SPSP: severity and prognosis score of PAP.
